# Accurate estimation of fractional vegetation cover for winter wheat by integrated unmanned aerial systems and satellite images

**DOI:** 10.3389/fpls.2023.1220137

**Published:** 2023-09-27

**Authors:** Songlin Yang, Shanshan Li, Bing Zhang, Ruyi Yu, Cunjun Li, Jinkang Hu, Shengwei Liu, Enhui Cheng, Zihang Lou, Dailiang Peng

**Affiliations:** ^1^ Key Laboratory of Digital Earth Science, Aerospace Information Research Institute, Chinese Academy of Sciences, Beijing, China; ^2^ International Research Center of Big Data for Sustainable Development Goals, Beijing, China; ^3^ School of Electronic, Electrical and Communication Engineering, University of Chinese Academy of Sciences, Beijing, China; ^4^ China Remote Sensing Satellite Ground Station, Aerospace Information Research Institute, Chinese Academy of Sciences, Beijing, China; ^5^ Information Technology Research Center, Beijing Academy of Agriculture and Forestry Sciences, Beijing, China

**Keywords:** fractional vegetation cover, winter wheat, UAS, remote sensing, machine learning

## Abstract

Accurate estimation of fractional vegetation cover (FVC) is essential for crop growth monitoring. Currently, satellite remote sensing monitoring remains one of the most effective methods for the estimation of crop FVC. However, due to the significant difference in scale between the coarse resolution of satellite images and the scale of measurable data on the ground, there are significant uncertainties and errors in estimating crop FVC. Here, we adopt a Strategy of Upscaling-Downscaling operations for unmanned aerial systems (UAS) and satellite data collected during 2 growing seasons of winter wheat, respectively, using backpropagation neural networks (BPNN) as support to fully bridge this scale gap using highly accurate the UAS-derived FVC (FVC_UAS_) to obtain wheat accurate FVC. Through validation with an independent dataset, the BPNN model predicted FVC with an RMSE of 0.059, which is 11.9% to 25.3% lower than commonly used Long Short-Term Memory (LSTM), Random Forest Regression (RFR), and traditional Normalized Difference Vegetation Index-based method (NDVI-based) models. Moreover, all those models achieved improved estimation accuracy with the Strategy of Upscaling-Downscaling, as compared to only upscaling UAS data. Our results demonstrate that: (1) establishing a nonlinear relationship between FVC_UAS_ and satellite data enables accurate estimation of FVC over larger regions, with the strong support of machine learning capabilities. (2) Employing the Strategy of Upscaling-Downscaling is an effective strategy that can improve the accuracy of FVC estimation, in the collaborative use of UAS and satellite data, especially in the boundary area of the wheat field. This has significant implications for accurate FVC estimation for winter wheat, providing a reference for the estimation of other surface parameters and the collaborative application of multisource data.

## 1 Introduction

Wheat is one of the main cereal crops in China, with its cultivated area and yield accounting for more than one-fourth of the total grain production (http://www.stats.gov.cn/). Timely and accurate acquisition of vegetation cover in2formation is particularly important for monitoring the growth status of wheat (Cheng, 2020; Wan et al., 2021; Abdelbaki and Udelhoven, 2022). Fractional vegetation cover (hereafter FVC) is usually defined as the vertical projection area of aboveground vegetation elements per unit of horizontal ground surface area (Li et al., 2023). It is a biophysical parameter related to the morphological structure and physiological characteristics of crops. To simulate regional or global wheat growth, crop canopy water content, biomass, and yield estimation, accurate FVC can directly calibrate crop models and hydrological models (Squire et al., 2021; Li et al., 2022b; Tenreiro et al., 2021; Wang et al., 2019; Zhu et al., 2019). It can also replace complex measurements of light interception capacity. It can also be used as an important parameter to determine other key physical quantities and plant chemical concentrations (De la Casa et al., 2018), for example, changes in leaf chlorophyll can cause significant differences in the canopy reflectance and transmittance spectra, and high-precision FVC can help to analyze changes in the canopy reflectance and improve estimates of chlorophyll content(Thenkabail and Lyon, 2016). As a holistic phenotypic characteristic that can investigate the vegetation status and farmland surface conditions across a range of scales, there is a widespread interest within the plant science and agronomy communities to attain high-throughput and precise quantification of FVC within a high spatiotemporal domain.

Satellite remote sensing has the advantages of long-time series, large scale (even global scale), and multi-scale (spatial and spectral scales), making it a common means of estimating FVC (Yu et al., 2021). Researchers have developed numerous FVC estimation methods based on coarse and medium spatial resolution satellite remote sensing data, including empirical models (Maurya et al., 2018; Riihimäki et al., 2019), linear unmixing models(Wang et al., 2020b; Wu et al., 2021), physical-based models (Jia et al., 2016; Tu et al., 2019), and machine learning (hereafter ML) methods(Graenzig et al., 2021; Maurya et al., 2022; Song et al., 2022). Among them, empirical models are generally considered easy to implement but have a limited scope of application (Niu et al., 2021), while linear unmixing models face challenges in extracting pure endmembers of different classes (Gao et al., 2020). Nowadays, through ML algorithms, even under the condition of unclear data distribution, nonlinear relationships between remote sensing images and vegetation information can be obtained (Zhang et al., 2022), which fits well with the complex characteristics of surface process remote sensing and is particularly suitable for research on remote sensing inversion (Ballesteros et al., 2020), fusion (Li et al., 2022a), downscaling (Liu et al., 2021) and other problems involving complex or unknown processes (Wang et al., 2019; Wang et al., 2020a; Lin et al., 2021; Xu et al., 2021).

Due to the limited number of spectral bands and high acquisition cost (Alvarez-Vanhard et al., 2021), high spatial resolution satellite data is still rarely used for estimating FVC in wheat. Therefore, there are currently two key challenges in estimating FVC based on low and medium-high spatial resolution satellite remote sensing data. Challenge 1 mainly refers to the need to improve spatial resolution while ensuring that the satellite remote sensing image has an appropriate number of spectral bands. For crops like wheat, whose growth activity mostly occurs in small areas, reliable FVC data with high temporal and spatial resolution is needed (Tao et al., 2021). Challenge 2 is that the current surface parameter inversion often requires field-measured data as reference, which often have a scale difference from the satellite images. The size of the vegetation survey plot usually ranges from 1 to 10^4^m^2^ (Walker et al., 2016; Riihimäki et al., 2019; Gu et al., 2021). Even with the acquisition of reference data for a single pixel, it typically necessitates sampling and numerous field observations, and frequently, it proves challenging to acquire copious ground samples while also avoiding destructive sampling.

In recent years, the swift progress of unmanned aerial systems (hereafter UAS) and photogrammetric techniques have unlocked fresh avenues for exploration in various research domains (Li et al., 2018; Riihimäki et al., 2019; Yan et al., 2019). Researchers have keenly discovered the powerful collaborative potential between UAS and satellite systems, which are widely used in the applications of ecology and precision agriculture (Yinka-Banjo and Ajayi, 2019; Alvarez-Vanhard et al., 2021). For example, in quantitative remote sensing research, numerical data obtained directly from UAS are utilized to calibrate a satellite-based model. Typically, the extracted values are surface (biophysical) parameters such as chlorophyll content or aboveground biomass, derived from spectral measurements (Zhang et al., 2019). Specifically in FVC estimation, UAS data are often used as sub-pixels to validate the satellite-derived FVC (hereafter FVC_satellite_), such as aiding in the enhancement of flood area estimates(Xia et al., 2017), observing the vegetation coverage of tundra under climate change (Riihimäki et al., 2019) or in the detection of invasive species (Graenzig et al., 2021). The strategy of mapping high spatial resolution images to coarse spatial resolution images through upscaling is also applied in satellite-to-satellite collaborative applications, such as accurately estimating the composition of shrub ecosystems by combining WorldView-2 (2m) and Landsat 8 (30m) images (Xian et al., 2015), and constructing regression models using GF-2 images (1m) and Landsat 8 surface reflectance as inputs to produce 30m spatial resolution FVC products (Song et al., 2022). Currently, some studies use coarse spatial resolution data to assist in the production of high spatial resolution FVC products, such as down-sampling Landsat FVC products using the random forest regression method(hereafter RFR) and using the UAS-derived FVC (hereafter FVC_UAS_) to accurately predict photosynthetic vegetation cover (Melville et al., 2019), using a recurrent neural network to increase the spatial resolution of GLASS FVC products from 500m to 250m (Liu et al., 2021), and using multi-resolution trees to produce spatiotemporal 30m spatial resolution FVC products by assisting Landsat 8 with MODIS data (500m) (Wang et al., 2020a).

In this paper, an accurate estimation of winter wheat FVC within the study region *via* ML techniques is proposed using medium spatial resolution satellite images and UAS images. The primary impetus driving this research is to bridge the scale gap between field and satellite images while advancing crop monitoring techniques through remote sensing methods. Our objective is to develop and assess a data-driven workflow. Firstly, based on preliminary experimental observations, two growth stages of wheat were chosen with the fast change stages in wheat coverage namely the Jointing Stage and the Booting Stage, to conduct “Ground-UAS-Satellite” synchronous experiments. Secondly, a strategy of Upscaling -Downscaling was applied to UAS and satellite data, respectively, to resample the scales to the ground sample level of 2 meters. Subsequently, conducted to UAS resampled images to generate the label data, which was then paired with the scaled satellite data to generate a training dataset. Finally, an ML model is conducted to accurately estimate the FVC_satellite_ of winter wheat. Finally, the FVC_satellite_ is evaluated by field-measurement data and analyses and discussion of the results.

## 2 Materials and methods

### 2.1 Study area

**Figure 1:**
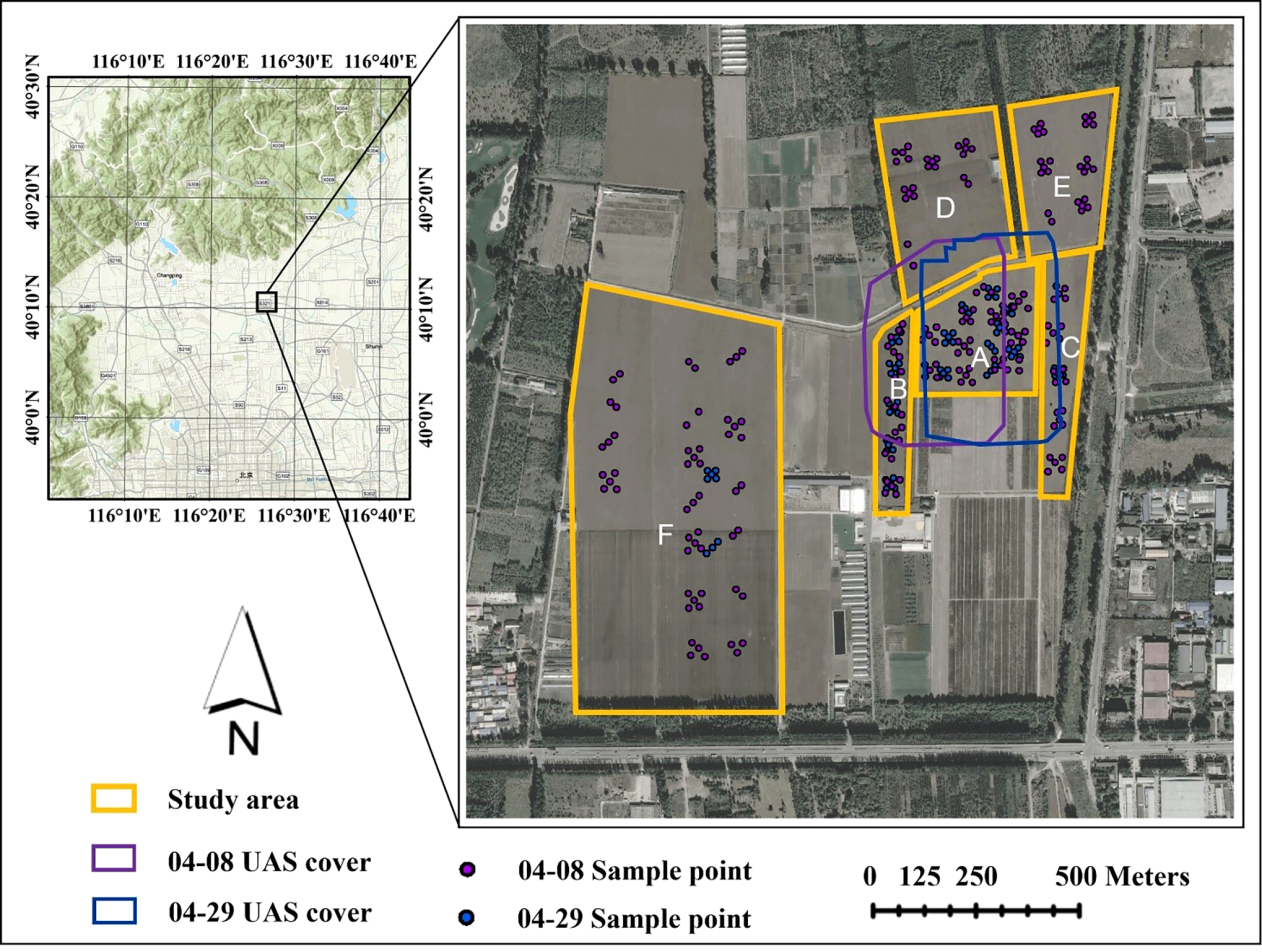
Geographic location of the study area. The topographical map at the upper left shows the location of the base in Beijing, China. The yellow boxes are winter wheat growing areas. The purple and blue boxes are the UAS image coverage, and the purple and blue points represent the locations of field sampling sites in the study area.

The study area is located within the Xiaotangshan National Precision Agriculture Research Demonstration Base in Changping District, Beijing (40°10’N, 116°26’E, 39 m). The terrain of the base is flat, and the climate is a warm temperate continental monsoon climate, with an average annual precipitation of 500-600 mm, which is a typical climate of the winter wheat area in the north. The total area of the base is 153.33 hectares (ha), and the area for winter wheat cultivation is 64.98 ha (**Figure 1**). Due to local regulations, the use of UAS is limited to a small area within the base (Purple and blue box in **Figure 1**), and accurate FVC estimation for the entire base or even larger areas requires satellite data support.

The study area is divided into six sections, labeled A to F in **Figure 1**. Plot A covers an area of 6.10 ha, and within it, experiments involving different water and fertilizer treatments, seeding densities, and irrigation methods were conducted. A section of the plot, in Plot A, was also designated as a no-intervention zone for winter wheat growth, which greatly increased the FVC diversity in the same period for winter wheat in the local area. Plots B to F represent normal winter wheat growth. Based on monitoring in previous years at the base, the growth stage for winter wheat was determined to be the jointing-booting stage when the variation of FVC is the greatest. As a result, there were two temporal phases to the “Ground-UAS-Satellite” synchronous observation experiments that were carried out for this study. The area covered by the UAS and satellite images on April 8 (Jointing Stage) and the location of the ground samples is marked in purple, while those on April 29 (Booting Stage) are marked in blue (**Figure 1**).

### 2.2 Data collection

#### 2.2.1 Unmanned aerial systems data and pre-processing

The UAS data were collected approach on April 8th and 29th, 2022. Upon confirming the revisit schedule of the Sentinel-2A/B satellite *via* SPECTATOR-EARTH (https://spectator.earth/), we carried out a synchronous ‘Ground-UAS-Satellite’ observational experiment in the presence of clear weather conditions.

The Parrot Sequoia agricultural multispectral camera, carried by both drone flights, was used to collect data. The camera consists of a multispectral sensor and an RGB sensor, which includes four 1.2 million-pixel single-band cameras and one 16 million-pixel RGB camera. The spectral parameters of the multispectral camera are shown in **Table 1**.

**Table 1:** Band parameters of multispectral sensor.

Band name	Wavelength (nm)	FWHM (nm)
Green	550	40
Red	660	40
Red edge	735	10
NIR	790	40

The UAS was equipped with a high-precision Real Time Kinematic module to provide real-time and centimeter-level positioning data, which were incorporated into the image metadata. We used a low, 40m flying altitude to achieve ultrahigh resolution imagery (RGB< 2.2 cm; Mul< 5.5cm), and an 85% frontal overlap between the image footprints (Riihimäki et al., 2019). We used the Pix4D Mapper (v. 4.5.6), which completes all the main structure-from-motion steps; including orthogonalizing the image and then correcting to surface reflectance using a standard whiteboard.

#### 2.2.2 Satellite data and pre-processing

With its multiple spectral channels, high revisit rates, and wide swath, Sentinel-2 provides real-time dynamic monitoring of the global environment and security (Li et al., 2022b; Putzenlechner et al., 2022). The Sentinel-2 images were gained from the Google Earth Engine of bottom-of-atmosphere reflectance that had been atmospherically corrected. In the visible spectral band, the spectral characteristics of wheat before maturity are dominated by various pigments in the leaves and stalks, with absorption valleys in the red band centered at 0.67 μm where chlorophyll strongly absorbs radiant energy. In the near-infrared (NIR) spectral band, the spectral characteristics depend on the cellular structure inside the leaf, and therefore, the NIR spectral band is often considered to be of high value for plant or non-plant differentiation. We chose three spectral bands of the Red, Red Edge, and NIR of Sentinel-2 as the data source to obtain the richest possible spatial information while meeting the spectral band requirements. The blue band (Band 2) data was not used because the blue band was considered easily contaminated by residual atmospheric effects (Jia et al., 2016), and the green band contributes to the biophysical change of vegetation and is beneficial to the remote sensing inversion of FVC. Due to partial cloud cover in the study area on 29 April, we finally chose two periods of data for the experiment, 8 April, and 2 May 2022.

#### 2.2.3 Field-measured data and pre-processing

The use of digital photography is the simplest and most reliable technique for testing and validating remote sensing information extraction (Liang and Wang, 2019), and it is widely used in crops ground measured FVC (hereafter FVC_real_) extraction (De la Casa et al., 2018; Wang et al., 2020a; Yu et al., 2021).

**Figure 2:**
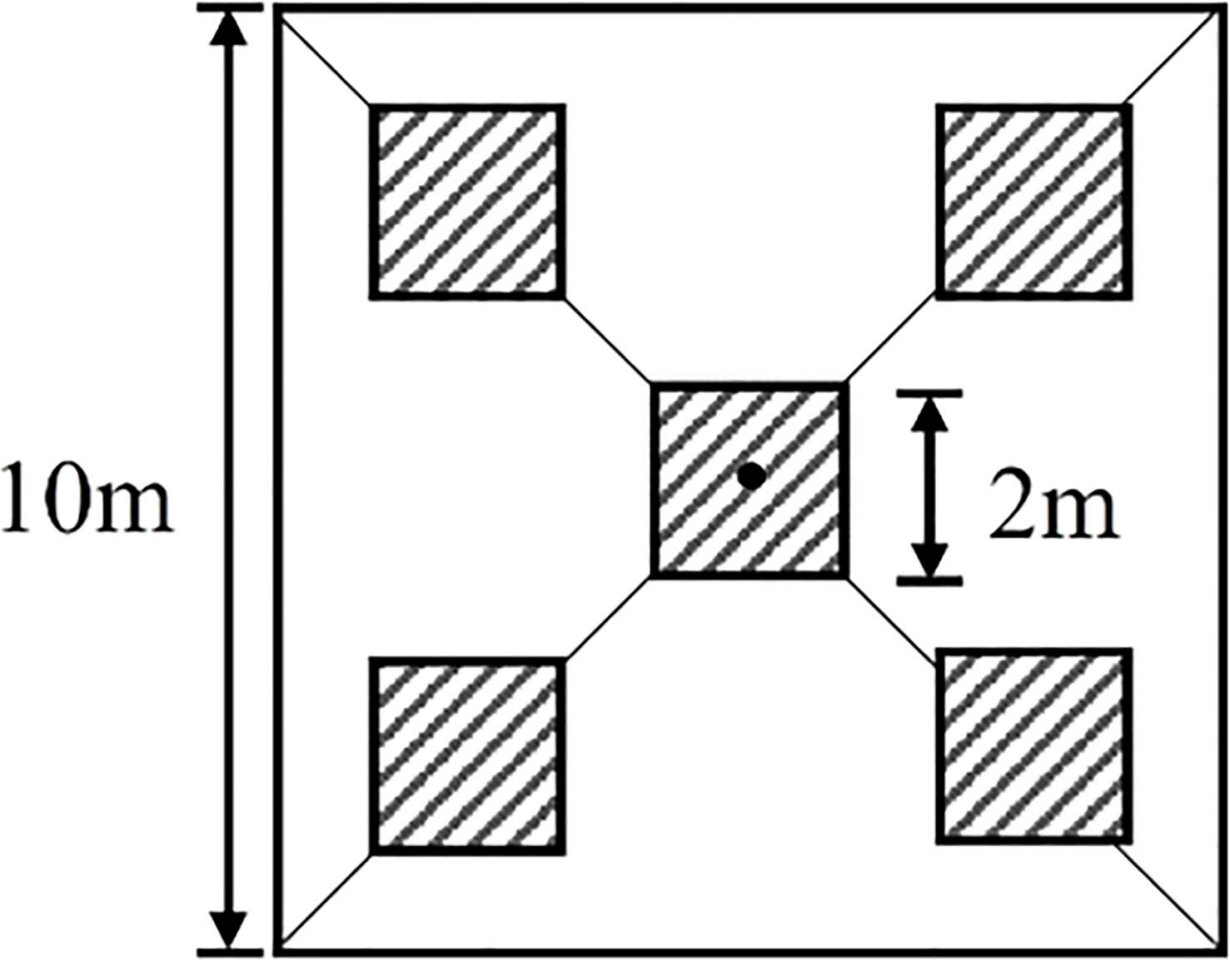
Strategies for measuring ground vegetation cover using digital cameras in 10m and 2m scale samples.

The ground sample point design used a five-point sampling method (**Figure 2**) (Yu et al., 2021). Five photographs (2m scale samples) were taken using a digital camera along two diagonals of the sampling point and their average was used as the FVC sample with a spatial resolution of 10m FVC_real_, while the FVC sample with a spatial resolution of 2m was only used for the middle of the 10m sample. So, 2m and 10m FVC_real_ have equal numbers. The choice of scales at 10m and 2m, respectively, was made based on their correspondence to the scales of satellite pixels and ground sample plots. The tool used to record the geographic coordinates of the center measurement point in each photo was the Tibbo A8 handheld GPS receiver, which achieved a positioning accuracy of 1m using the Satellite-Based Augmentation System. To further improve the positioning accuracy, we created a square polygon (2m) around the central coordinates in ArcMap 10.6 and used the Georeferencing tool to manually align it with the RGB data from the UAS to obtain the final positioning accuracy (<0.41m). The number of ground samples collected in each of the two time periods is shown in **Table 2**.

**Table 2:** Ground sampling of the experiments.

Date	Total	Number of samples in the area covered by UAS images				Number of samples in the area not covered by UAS images				
		Plot A	Plot B	Plot C	Plot D	Plot A	Plot B	Plot C	Plot D	Plot E/F
4-8	210	52	0	6	1	0	22	24	23	82
4-29	77	34	17	0	0	5	7	6	0	8

In this manuscript, Gaussian fitting and segmentation algorithms are utilized to extract FVC from the image (Liu et al., 2012; Yu et al., 2021). The core of the algorithm is to convert the color space of a digital image from RGB to CIE L*a*b*. At this point, the histogram of the a* channel presents two approximate Gaussian distributions, and the following function is employed to analyze the distribution of the transformation values:


(1) $$f\left(x\right)=\frac{w_{1}}{\sqrt{2\pi }\delta_{1}}e^{\frac{{\left(x-\beta_{1}\right)}^{2}}{-2\delta_{1}^{2}}}+\frac{w_{2}}{\sqrt{2\pi }\delta_{2}}e^{\frac{{\left(x-\beta_{2}\right)}^{2}}{-2\delta_{2}^{2}}}$$

where $\beta_{1}$ and $\beta_{2}$ are the means of the winter wheat and the background, respectively; $\delta_{1}$ and $\delta_{2}$ are the standard deviations of the winter wheat and the background, respectively; and $w_{1}$ and $w_{2}$ are the weights of the winter wheat and the background, respectively.

**Figure 3:**
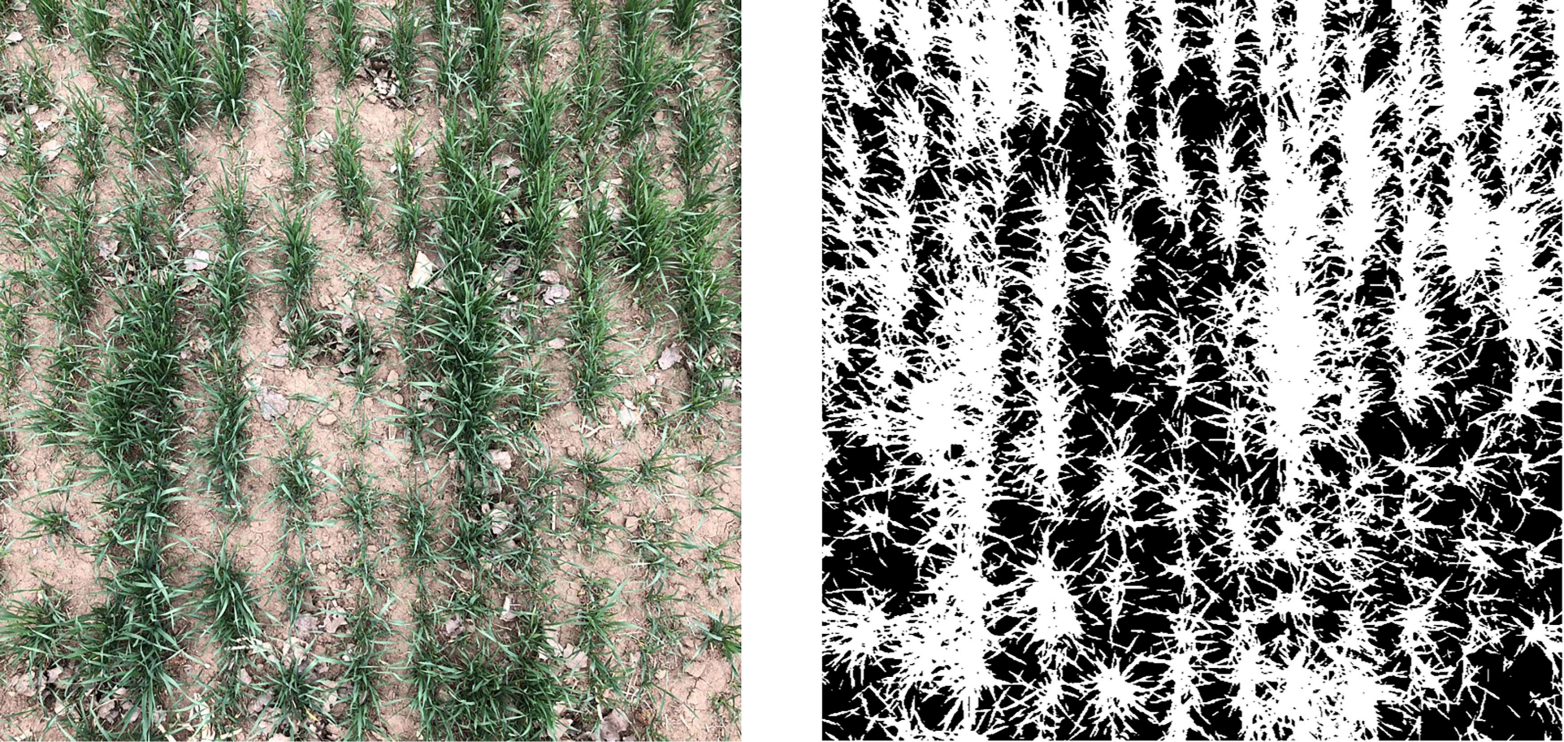
The examples of extracting FVC from the field-measured images.

Finally, a threshold is determined to segment the two Gaussian distributions and fit the curves of the two parts for unbiased estimation of FVC. The original digital photograph (left) and the algorithmically processed segmentation map are shown in **Figure 3**, resulting in the FVC in this 2m sample. It is worth noting that in practice it is difficult to determine the threshold for plots that appear to be wet with water, and often a customized threshold is required by visual judgment. The Gaussian fitting and segmentation algorithms were implemented entirely in MATLAB 1.6.

### 2.3 Methodology

#### 2.3.1 Workflow for accurate estimation of winter wheat FVC

**Figure 4:**
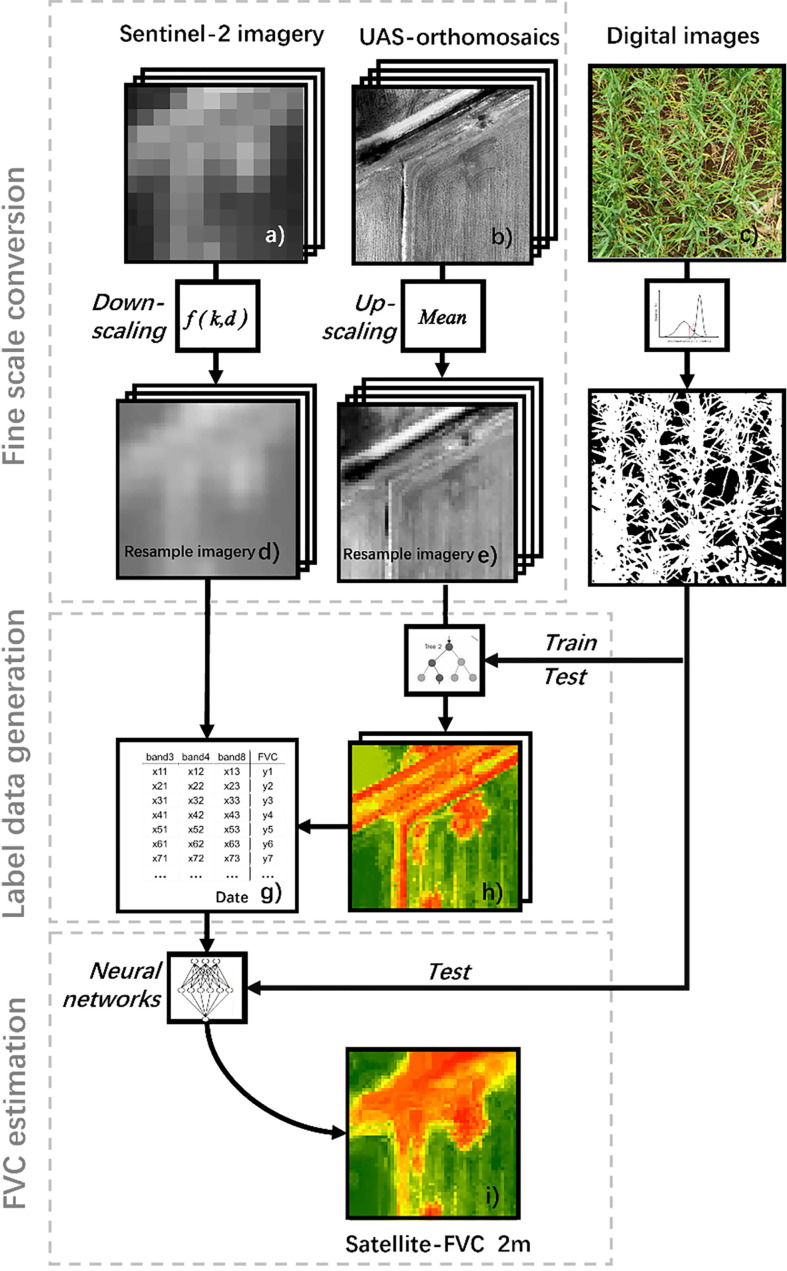
Workflow for inversion of winter wheat FVC using Ground-UAS- Satellite data and ML models. **(A)** satellite image from the region of interest. **(B)** UAS image from the region of interest. **(C)** ground measurement photograph.**(D)** downscaled satellite image. **(E)** upscaled UAS image. **(F)** Field-measured FVC. **(G)** FVC_UAS_ extracted from UAS. **(H)** training data pair consisting of downscaled satellite images and FVC_UAS_. **(I)** predicted FVC_satellite_ of the region of interest. where **(A, B, D, E, H, I)**, and **(I)** show images of the same location.

To accurately estimate FVC for winter wheat, we integrated UAS, and satellite imagery based on the ML algorithm. First, conduct ground, UAS, and satellite experiments simultaneously to collect data (**Figures 4A–C, F**). Use the Strategy of Upscaling-Downscaling to perform scale conversion operations on UAS and satellite reflectance data (**Figures 4D, E**), completing data preprocessing. Then, use the optimized regression models to estimate FVC_UAS_ (**Figure 4H**) using UAS data and field-measured data in plot A (**Figure 1**). Evaluate the model with the highest accuracy using independent validation data and obtain a large amount of labeled data. Finally, pair the downscaled satellite data with the labeled data to obtain training data (**Figure 4G**), train the BPNN model, and obtain accurate FVC for wheat based on satellite data (**Figure 4I**).

#### 2.3.2 Fine-scale conversion

Scale is one of the fundamental and important issues in remote sensing (Li and Wang, 2013; Liu et al., 2018), Establishing the conversion relationship of surface parameters from one scale to another, including both upscaling and downscaling aspects. However, existing research on FVC space scale conversion is mainly focused on upscaling (Riihimäki et al., 2019; Lin et al., 2021; Song et al., 2022). To obtain more accurate FVC, we adopt a strategy of unifying satellite and UAS images to the ground plot (2m) scale, supported by the high spatial resolution of UAS imagery and the powerful learning capability of machine learning. Based on the findings of other researchers and our observations of the winter wheat growth process in experiments, the canopy characteristic scale (Tang and Liu, 2014) of winter wheat at different FVC stages is not consistent, which leads to a continuously changing appropriate observation scale (Jones and Vaughan, 2010). The canopy characteristic scale is the basis of research object linear and non-linear mixed, and it is the premise of the optimal scale of observation objects. From the perspective of ray radiation transport, there is a characteristic scale nonlinear mixed into the linear mixed transition. Incident radiation between the transition characteristic scales of the object is independent of the optical properties, and it can more effectively describe the canopy group. By selecting the right scale, one can produce remote sensing data with a twofold increase in efficiency. Since the average wheat row spacing in actual observations is 0.151m ( ± 0.003m) and considered the accuracy after GPS registration, we subjectively defined the target scale as 2m. In fact, the 2 m scale is not set in stone, and it is interesting to investigate the best observational scale for a particular crop. As a result, we may improve our scaling research in future studies.

Downscaling of satellite images

A common method of downsizing images involves reducing the resolution of high-resolution images to low-resolution images, followed by using an interpolation method to reconstruct high-resolution images. Popular interpolation methods include nearest neighbor (Jiang and Wang, 2015), bilinear (Kirkland and Kirkland, 2010), and bicubic interpolation (Keys, 1981). The cubic convolution interpolation algorithm is a widely used method, which constructs the interpolation basis function by operating on the gray values of the 16 adjacent points within a 4 × 4 neighborhood of the target point and then uses the function to reconstruct the coarse-resolution image. W(x) , the interpolation basis function, is the best theoretical cubic approximation in theoretical of function inc(x)=(sinx)/x , and is shown in (2).


(2) $$W\left(x\right)=\{\begin{array}{c} {\left|x\right|}^{3}-2{\left|x\right|}^{2}+1,\;\;\;\;\;\;\;\;\;\;\;\;\;for\left|x\right|<1\;\;\;\;\;\\ -{\left|x\right|}^{3}+5{\left|x\right|}^{2}-8\left|x\right|+4,\;for\;1\leq \left|x\right|\leq 2\\ 0,\;\;\;\;\;\;\;\;\;\;\;\;\;\;\;\;\;\;\;\;\;\;\;\;\;\;\;others\;\;\;\;\;\;\;\;\; \end{array}$$

which x is the raw pixel value (the reflectance value of Sentinel-2 L2A data) of the target point.

f(i+u,j+v) represents the pixel value of the target point (i+u,j+v) in the source image, and the interpolation formula is as follows:


(3) f(i+u,j+v)=ABC

The forms of matrix A,B,C are as follows:


(4) A=[W(u+1)W(u)W(u−1)W(u−2)]

(5) B=f(i−1:i+2,j−1:j+2)

(6) C=[W(v+1)W(v)W(v−1)W(v−2)]

where the distances between the target point and the nearest point (i,j) of the source image are u and v , respectively.

The cubic convolution interpolation algorithm can not only maintain good image details but also effectively suppress artifacts and noise, which is why it is widely used in image downsampling reconstruction (Lepcha et al., 2023). The antialiasing attribute of tricubes convolution interpolation is also a mainstream method for constructing super-resolution training sets, which is widely used in pre-up sampling super-resolution models or directly for satellite surface reflectivity reconstruction (Li et al., 2020; Lepcha et al., 2023). Here, Sentinel-2 images were processed by cubic convolution interpolation algorithm to achieve fine-scale conversion scale conversion (**Figures 4A, D**).

Upscaling of UAS images

Downscaled satellite images were used to build a raster in ArcMap 10.6 and used the *Zonal* tool to aggregate the UAS multispectral imagery to achieve the upscaling of UAS data (Riihimäki et al., 2019) (**Figure 4E**). It is worth mentioning that after resampling the 10m pixels to 2m pixels using cubic convolution, the original pixel values (10m) are preserved in the middle of the resampled 2m pixels.

#### 2.3.3 Extract FVC_UAS_ using UAS data

To obtain label data with the highest possible accuracy, we have improved the four methods for extracting FVC_UAS_, which are the RFR (Breiman, 2001), the Normalized Difference Vegetation Index image-based dichotomous model (hereafter NDVI-based) (Xiao and Moody, 2005), and the support vector regression (hereafter SVR) (Lin et al., 2021) using multispectral data, and a half-Gaussian fitting method (hereafter HAGFVC) (Li et al., 2018) using RGB data, respectively.

Random forest regression model

RFR is an ensemble learning algorithm composed of multiple regression trees, which models the relationship between band reflectance and FVC using a set of decision rules using RFR. Each regression tree generates a prediction value, and the final predicted value of RFR is the average response value of all regression trees. The core algorithm of RFR is as follows:


(7) $$\underset{M,\;s}{\underset{︸}{\min}}\left[\underset{N_{1}}{\underset{︸}{\min}}\sum_{X_{i}\in \;K_{1}\;(M,\;s)}{\left(y_{i}-N_{1}\right)}^{2}+\underset{N_{2}}{\underset{︸}{\min}}\sum_{X_{i}\in \;K_{2}\;(M,\;s)}{\left(y_{i}-N_{2}\right)}^{2}\right]$$

Where $N_{1}$ and $N_{2}$ is the sample output mean of $K_{1}$ and $K_{2}$ data set, M is the division feature and s is the division point, $y_{i}$ the i-th sample point.

Two parameters need to be optimized in RFR: the number of trees in the random forest (Ntree) and the number of bands randomly extracted at each node (Mtry). The range of Mtry is set from 1 to 31 with a step size of 1, and the range of Ntree is set from 100 to 2,000 with a step size of 100. After 10-fold cross-validation, the optimal Mtry and Ntree values were found to be 2 and 350, respectively. To enhance the model’s generalization ability, ground measurement data from two temporal phases were used together to train and test the model, with a training-to-validation data ratio of 8:2, as was done for the SVR and NDVI-based methods.

Support vector regression model

SVR is an ML regression algorithm that is derived from the idea of support vector machines (SVM) by introducing insensitive loss coefficients. Given the kernel function $K\left(x_{i},x_{j}\right)$ , SVM can be solved using the method of solving linear problems to solve non-linear problems in the original input space. Here, we are using the Radial basis kernel function. The optimal hyperplane is defined in the space spanned by the kernel function of the support vectors $x_{i}$ :


(8) $$f\left(x\right)=\sum_{i=1}^{n}\left(\alpha_{i}K\left(x_{i},x_{j}\right)\right)+w_{0}$$

In the equation, $x_{i}$ represent the support vector, n is the number of support vectors, $\alpha_{i}$ is the basic coefficient corresponding to the support vector, which is mainly affected by the penalty coefficient, and $w_{0}$ is the absolute coefficient.

Similar to the optimization method in the reference literature, the grid search method is used to optimize the kernel parameter (gamma) that reflects the distribution of samples in the feature space, and the penalty coefficient(cost) that affects the complexity and stability of the model (Lin et al., 2021). After 10 rounds of cross-validation, the optimal gamma and cost values were determined to be 0.8 and 3, respectively.

Pixel dichotomy model

The binary pixel model is currently the most widely used method for estimating FVC. It postulates that the pixel information received by satellite sensors is composed of vegetation and soil, with FVC representing the percentage of vegetation-occupied pixels. NDVI is considered to be a good indicator of FVC. Therefore, this study utilizes a pixel binary model with NDVI as the input parameter to estimate FVC in wheat. The formula is as follows:


(9) $$FVC=\frac{NDVI-NDVI_{S}}{NDVI_{V}-NDVI_{S}}$$

where $NDVI_{S}$ , and $NDVI_{V}$ are NDVI values in the area that were completely covered by soil and vegetation, respectively.

HAGFVC model

The principle behind HAGFVC is to fit two half-Gaussian distributions in the International Commission on Illumination (CIE) Lab* color space, and then determine the threshold based on the parameters of the Gaussian distribution to generate a more accurate FVC estimate. As it directly extracts FVC using UASs’ RGB data, its workflow first extracts FVC and then performs a scale transformation into a 2m upscaling operation, and finally evaluates the extracted FVC results using the field measurement data of plots B, C, and D.


(10) $$h\left(x\right)=\frac{1}{\sqrt{\left(2\pi \right)\sigma }}exp-\frac{{\left(x-\frac{\mu }{\sigma }\right)}^{2}}{2}\;\;\;\;\;x\geq \mu_{b},x\leq \mu_{v}$$

Where h(x) is the half-Gaussian distribution function; μ and σ are the mean value and standard deviation, respectively; subscripts v and b refer to vegetation and background.

After using this method to separate vegetation from the background, evaluate the model accuracy using the square polygons established in ground measurement data in ArcMap 10.6. A MATLAB GUI tool developed by the authors of the method was used for implementing the HAGFVC method.

FVC_UAS_ accuracy verification

The independent validation dataset for the FVC_UAS_ estimation model was composed of FVC_real_ from plots B, C, and D that had UAS data coverage (**Figure 1**). To evaluate the accuracy of the FVC estimation model, the study uses the coefficient of determination decision factor (R^2^) and root mean square error (RMSE) to measure the model’s predictive performance. The formulas for calculating R^2^ and RMSE are as follows:


(11) $$R^{2}=\frac{SSR}{SST}$$

(12) $$RMSE=\sqrt{\frac{1}{n}\sum_{i=1}^{n}{\left(y_{i}^{'}-y_{i}\right)}^{2}}$$

#### 2.3.4 Accurate FVC_satellite_ estimation based on neural networks

The neural network learns from a training dataset by imitating human learning capabilities to establish relationships between variables that are robust to noisy data and can approximate multivariate nonlinear relationships (Rumelhart et al., 1986). Back propagation neural network (hereafter BPNN) is one of the most widely used models in artificial neural networks. It has been applied to estimate basic vegetation variables such as FVC (Jia et al., 2016), FAPAR (Putzenlechner et al., 2022), and aboveground biomass (Zhu et al., 2019), and has been proven to be an effective algorithm. BPNN consists of three parts: input layer, hidden layer, and output layer, with its core being the adjustment of synaptic weights to achieve overall error below the expected threshold. Therefore, this study has selected BPNN to construct an FVC estimation algorithm. BPNN can learn from the training dataset and create relationships between reflectance under different surface conditions and FVC. The trained BPNN can then provide the optimal FVC estimate based on the actual reflectance of remote sensing data.

The BPNN used in this study took three Sentinel-2 surface reflectance bands, specifically green (Band 3), red (Band 4), and NIR (Band 8), as input. The output of the model was the corresponding FVC values for the three surface reflectance bands. The hidden layer of the BPNN contained 6 nodes, with “sigmoid” and “linear” functions used as activation functions for the hidden and output nodes, respectively. The Levenberg-Marquardt minimization algorithm was used to calibrate the synaptic weights during the training process. The learning dataset consisting of input-output pairs from FVC_UAS_, and satellite data were randomly divided into three parts: 80% of the training data was used to train the BPNN, and 20% of the training data was used to test the convergence process of the model. The performance threshold was set at RMSE with a value of 0.06 as the indicator during the training process, with a maximum iteration of 5000 times. Eventually, the expected goal was achieved after 727 iterations in the 2m-scale training, while the performance did not reach the predetermined goal in the 10m-scale training. Therefore, the model that showed RMSE continuously for 15 iterations without further changes was selected as the final training model.

Traditional and advanced estimation methods, including the NDVI-based method, the optimized ML method RFR, and the deep learning method LSTM (Yu et al., 2021) were used to compare with the BPNN method, where the optimization strategy of RFR is consistent with that in the FVC_UAS_ estimation process, and the optimization strategy of LSTM model was to adjust the number of hidden layers, learning rate, and batch size.

FVC_satellite_ accuracy verification

The independent validation dataset for the 2m scale FVC_satellite_ estimation model was composed of ground-truth data from plots B to F and FVC_real_ from plot A during the tasselling stage that was not covered by UAS data, while the validation method in the 10m scale FVC estimation is tenfold cross-validation instead of independent validation. Here, the model’s predictive performance was still measured using the coefficient of determination decision factor and root mean square error.

## 3 Results and discussion

### 3.1 Accuracy assessment of FVC_UAS_

**Figure 5:**
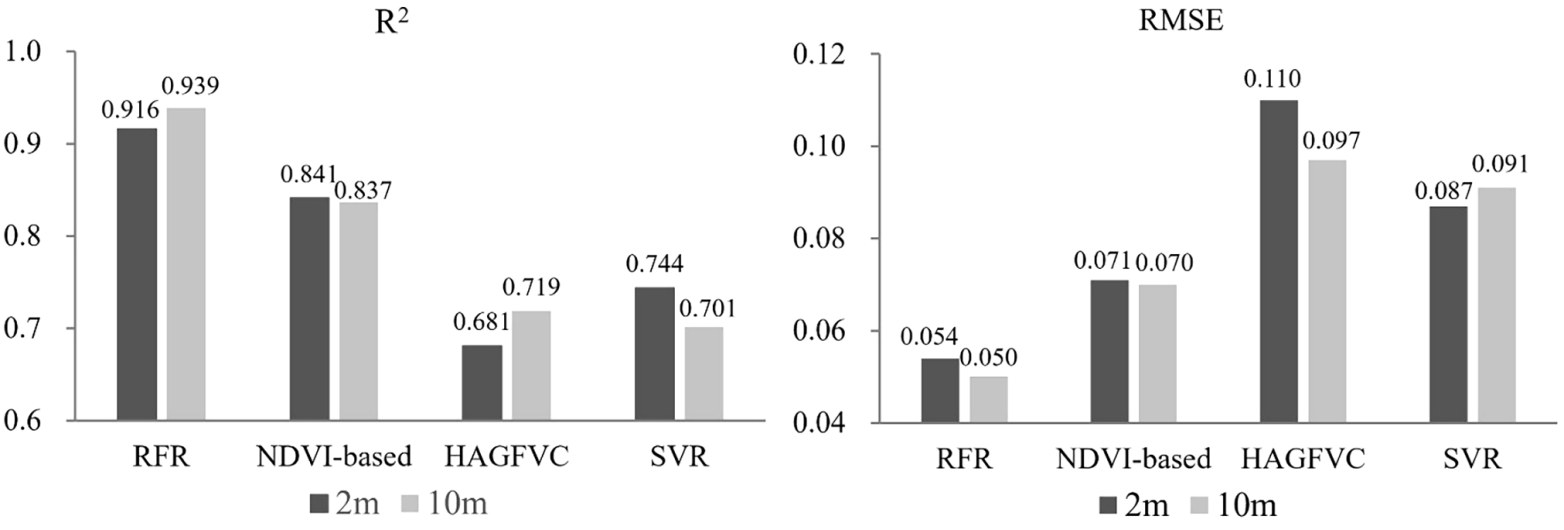
Comparisons of the RMSE and R^2^ of different FVC_UAS_ estimation models.

As FVC_UAS_ serves as an input for neural network models estimating FVC derived from satellite data, the FVC_UAS_ must be of the highest possible accuracy. The inversion results of the four FVC_UAS_ extraction models indicate that the RFR model driven by UAS multispectral data has the highest accuracy at the 2m and 10m scales. The ten-fold cross-validation average prediction values of the model are highly fitted with the reference values, with an R^2^ index of 0.9359 and an RMSE of 0.0544 (**Figure 5**). In comparison, the commonly used NDVI-based method has an R^2^ of 0.8405 and an RMSE of 0.0765, representing an 11% increase in R^2^ and a 29% decrease in RMSE. The other two models performed poorly.

**Figure 6:**
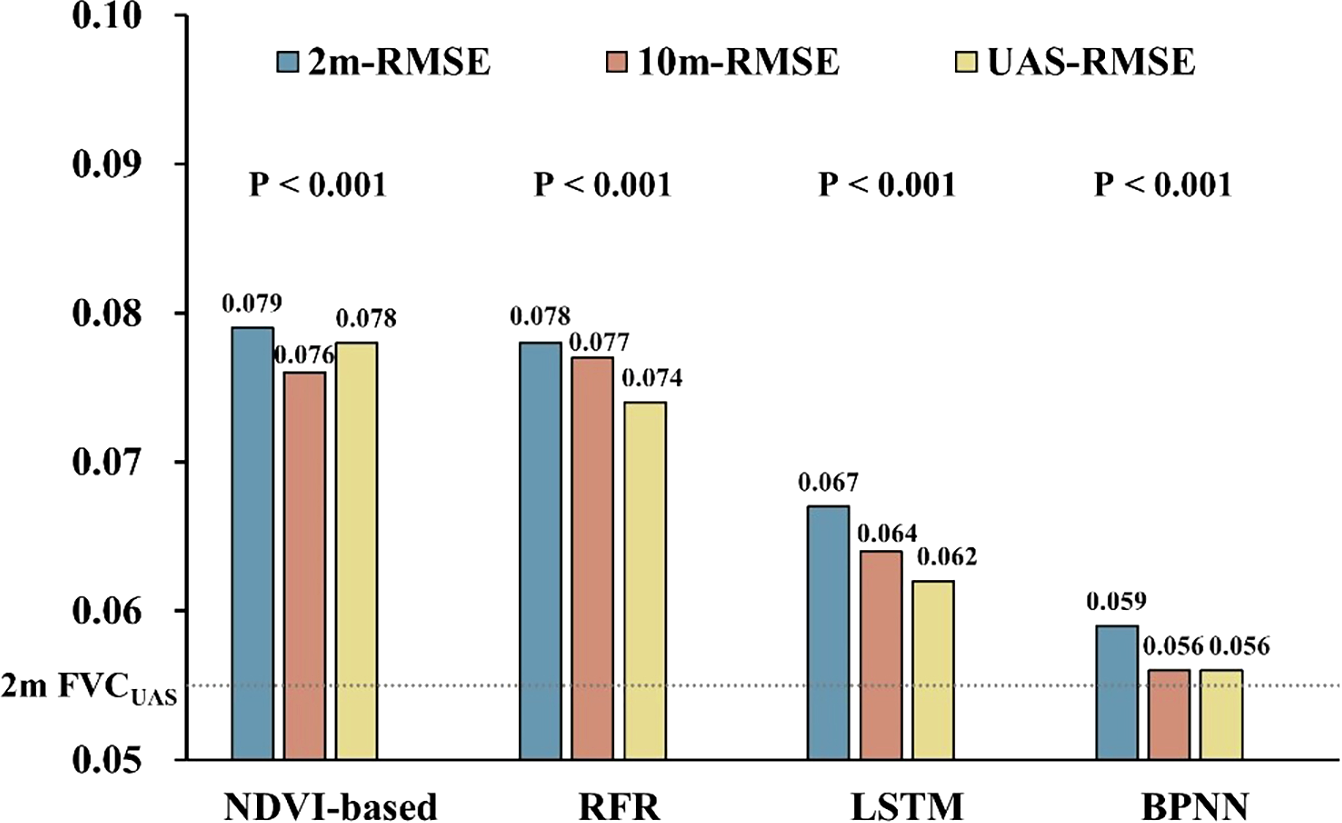
Comparison of accuracy among different FVC extraction models; The vertical axis is the RMSE value; The dashed line represents the results fitted by 2m FVC_UAS_ and FVC_real_.

**Figure 7:**
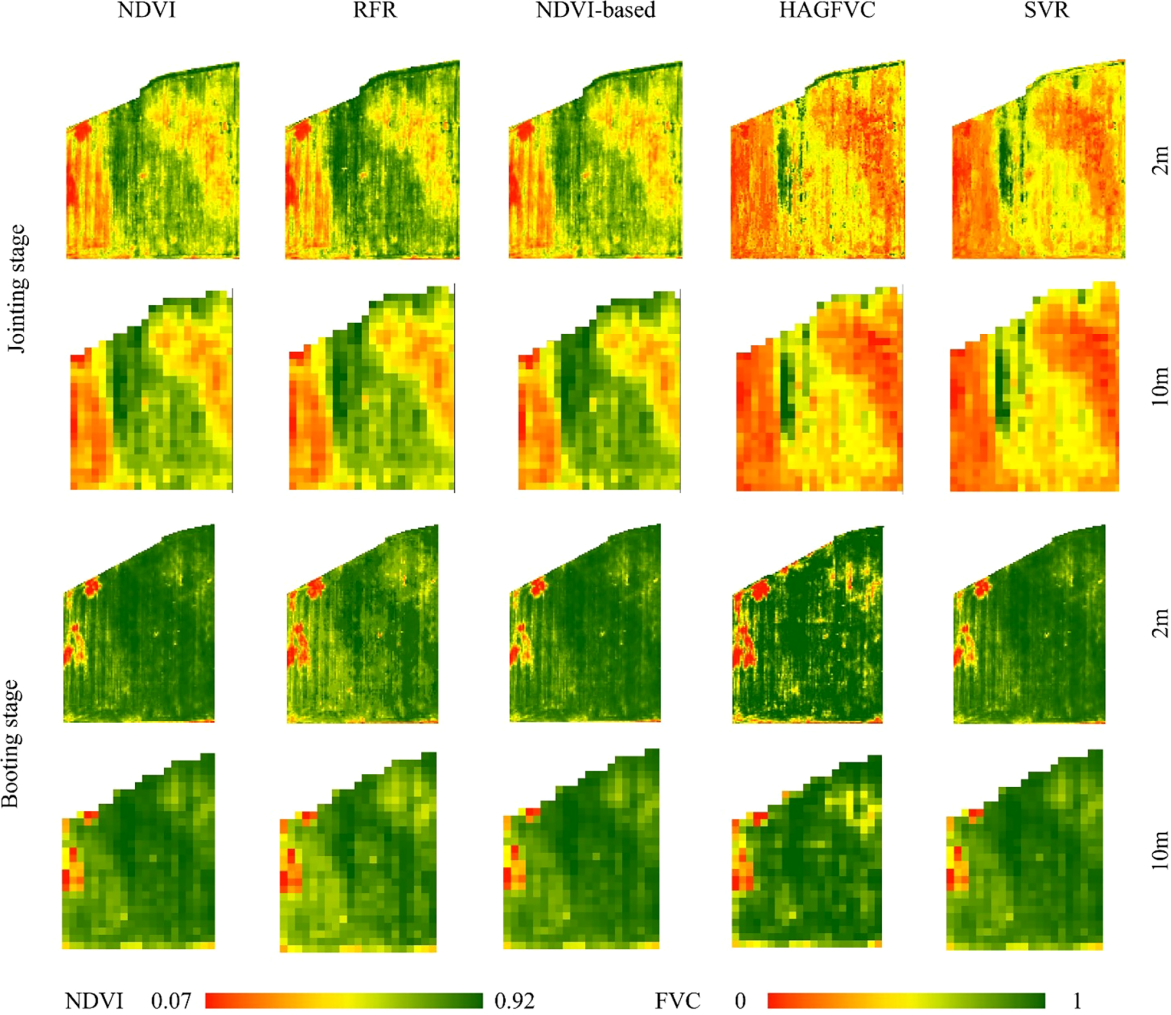
The examples of extracting FVC_UAS_ from the UAS images in plot A.

From **Figure 6**, it can be observed that during the jointing and booting stages, the RFR and NDVI-based inversion results are consistent with the distribution of NDVI. This is consistent with previous research which found a strong correlation between FVC and NDVI (Jia et al., 2016; Gao et al., 2020; Song et al., 2022). Moreover, during the jointing stage, RFR is still able to accurately retrieve FVC in areas with high FVC, while NDVI-based and NDVI maps show a ‘saturation phenomenon’. During the booting stage, when FVC is generally high, NDVI-based, SVR, and HAGFVC all show varying degrees of overestimation. This further confirms the characteristic of the decreased sensitivity of NDVI in areas with high FVC. The HAGFVC and SVR models often estimate lower FVC during the jointing stage and higher FVC during the booting stage. The reason for this difference is different for the two models. For HAGFVC, its principle is similar to that of the mixed pixel decomposition model (Gao et al., 2020). However, the 2cm spatial resolution of the UAS is still relatively coarse for wheat leaves, resulting in overmuch mixed pixels and difficulties in extracting pure pixels, which is often considered the main reason why such models are limited in their use. The inversion results of HAGFVC at the 10m scale are better than those at the 2m scale (**Figure 7**), and this difference should be due to the accuracy of the GPS handheld used in the FVC_real_ measurements. Finer scales should be equipped with higher-precision positioning instruments. The performance of SVR is not satisfactory, presumably due to insufficient optimization of gamma and cost values. In addition, during the optimization process of RFR, it was found that the absence of any one to three of the four spectral bands of the UAS multispectral sensor or their combinations would reduce the prediction accuracy. This suggests that all four bands of the Parrot Sequoia have a strong positive correlation with FVC.

Overall, the inversion results of the RFR model were utilized for label data production, generating a total of 27,817 2m-scale label data and 1,076 10m-scale label data for winter wheat in the jointing and booting stages, respectively, as shown in **Table 3**. The data from both growth stages were used together to train an accurate FVC estimation model to further enhance the robustness of the model.

**Table 3:** Amount of tagging data for different growth periods of winter wheat.

Growth periods	Number of UAS samples	
	2m	10m
Jointing Stage	15530	602
Booting Stage	12287	471

### 3.2 Comparisons of different FVC_satellite_ estimation models

#### 3.2.1 Parameter settings for different FVC_satellite_ estimation models

All FVC_satellite_ extraction models were optimized for optimal performance. In the FVC_satellite_ estimation using the Strategy of Upscaling-Downscaling, the optimized LSTM network had 3 layers with 100 hidden neurons in each layer. The dropout rate for each dropout layer was set to 0.3, the training epoch was set to 550, the batch size was set to 128, and the learning rate was set to 0.0005. For the LSTM and BPNN models, 27,817 sample data were divided into training data, validation data, and testing data in an 8:1:1 ratio. The key parameter NREE for the optimized RFR was set to 500, and Mtry was set to 2. The training and testing data ratio for RFR and NDVI-based methods was 8:2. It is worth noting that the reason why 10m FVC_satellite_ was still available in this strategy is that we upscaled 2m FVC_satellite_ to 10m (10m-RMSE, **Figure 6**) using the *Zonal* tool in ArcMap. In the FVC_satellite_ estimation using the strategy of upscaled operations on UAS data only, the parameters of the BPNN model and the LSTM model remained unchanged, and the RFRs’ optimal Mtry and Ntree values were found to be 1 and 300.

**Figure 8:**
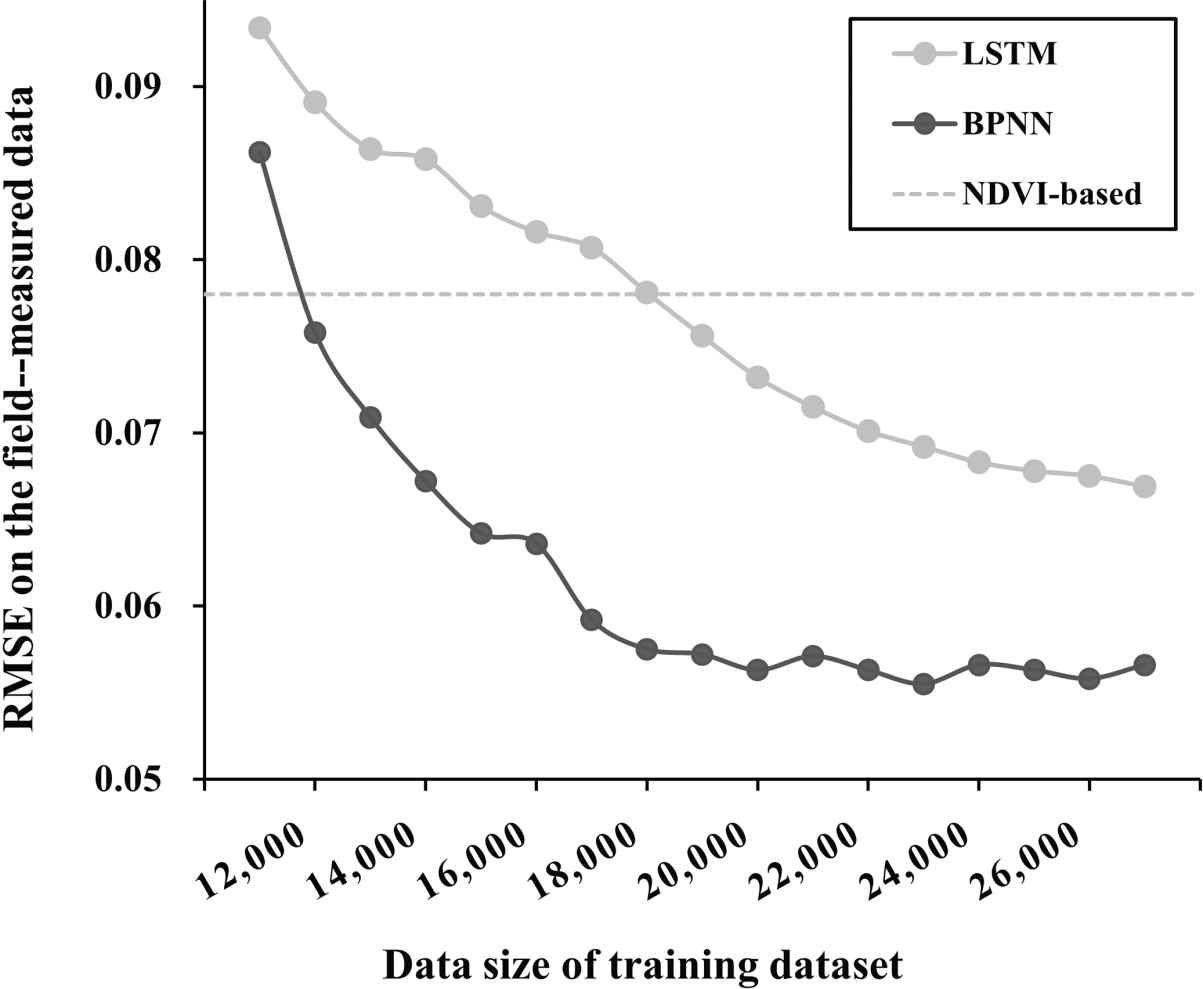
RMSE of the BPNN and the LSTM method on the field measured data under different data sizes of the simulated training dataset.

The neural network models’ network structure and multi-layer learning mechanism can extract deep and abstract features of data. However, because of this, these models are sensitive to the amount of training samples. A large amount of training data can ensure the model’s prediction accuracy, but too much data may bring problems such as information redundancy and long training time. Therefore, a quantitative analysis of the relationship between data volume and model accuracy is needed to ensure both model accuracy and training efficiency with sufficient data (Lin et al., 2021; Yu et al., 2021). In our designed experiment, we randomly selected 0-27000 samples (with a 1000 interval) from 27817 sample data to test the performance of the model. The results showed that BPNN and LSTM models converge when the data volume is more than 12000. Meanwhile, because the NDVI-based model’s prediction results are relatively stable when inputting 12000 or more data, we took the average of the results of 10 repeated experiments as a reference (**Figure 8**, Gay line). The results showed that when the training data volume reached 15000, the prediction accuracy of both BPNN and LSTM models could stably exceed that of the NDVI-based model. When the training data volume reached 20000, the prediction accuracy of the BPNN model tended to be stable. However, for the LSTM model, it seems that the full performance of 27817 sample data cannot be explored. If more sample data is input, there is still further improvement in its prediction accuracy.

#### 3.2.2 Comparison of accuracy of FVC_satellite_ estimation models

The proposed method (BPNN) was evaluated using reliable 2m FVC_real_, 10m FVC_real_ and 2m FVC_UAS_. Compared to NDVI-based, RFR, and LSTM models, the average RMSE of BPNN models’ inversion results were reduced by 25.3%, 24.4%, and 11.9%, respectively, after fitting with 2m FVC_real_ (2m-RMSE, **Figure 6**). At the same time, the BPNN inversion results were verified by 2m FVC_UAS_, showing the same trend (UAS-RMSE). Interestingly, upscaling the prediction results from a 2m scale to a 10m scale resulted in a general decrease in RMSE when tested against the independent validation dataset of 10m FVC_real_ (10m-RMSE). To further explore the differences between the two best-performing methods, BPNN and LSTM, we tested their data sensitivity. The results showed that when the training data volume reached 20,000, the prediction accuracy of BPNN tended to stabilize. However, for the LSTM model, 27,817 sample data seemed insufficient to fully exploit its performance.

The average RMSE of the BPNN model (0.059) is significantly close to the average RMSE of 2m FVC_UAS_ (0.054) compared to other models. The UAS-RMSE of all four models is lower than the 2m-RMSE, which we believe is the result of error propagation because the error between 2m FVC_UAS_ and FVC_real_ is still preserved between 2m FVC_satellite_ and FVC_real_ and cannot be eliminated with the current amount of data. Similar examples of error propagation should be avoided at the experimental design stage, such as direct validation using UAS data (Riihimäki et al., 2019) or direct validation using high spatial resolution data (Wang et al., 2020a; Song et al., 2022). The RFR model can achieve good accuracy in predicting FVC_UAS_, but when using FVC_UAS_ and satellite data as training data and 2m FVC_real_ as validation data, its RMSE is close to the fitting result of the NDVI-based model, while when using 2m FVC_UAS_ as validation data, its RMSE is 5.4% lower than the fitting result of the NDVI-based model. We believe that RFR can still effectively solve the problem of multicollinearity between independent variables compared to traditional NDVI-based models, but its learning ability is limited when the relationship between input variables and the modeling target is more complex. As many previous works have reported, the performance of RFR in complex nonlinear relationships is not ideal (Wang et al., 2019; Cheng et al., 2022; Hu et al., 2022). The main reason why all 10m-RMSE are lower than 2m-RMSE is that the RMSE of 10m FVC_UAS_ is lower than that of 2m FVC_UAS_, which explains the same trend even for the traditional NDVI-based model.

#### 3.2.3 Comparison of estimation accuracy of different models for uni-temporal FVC_satellite_

**Figure 9:**
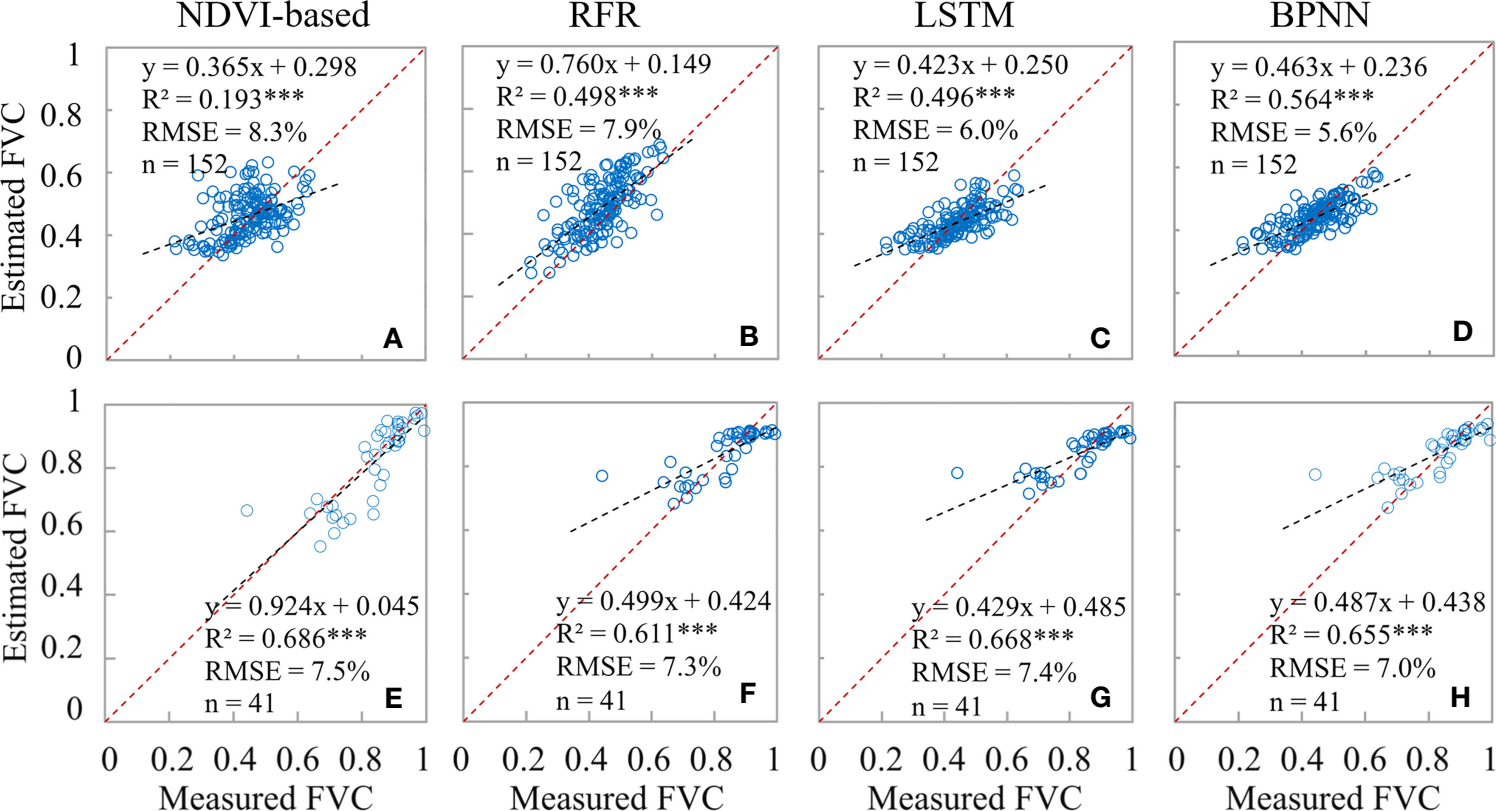
Comparison of the accuracy of different FVC_satellite_ extraction models. **(A–D)** are the results of winter wheat at the Jointing Stage; **(E–H)** are the results of the Booting Stage.

The establishment of the model involved the use of two phases of FVC_UAS_ data, which not only enhances the robustness of the model but also enables the estimation of winter wheat FVC in a single time phase. As shown in **Figure 9**, it can be observed that the RMSE of the BPNN and LSTM models based on neural networks is consistently lower during the jointing period than during the booting period. This difference can be well explained by the proportion of FVC_real_ (53:34) and FVC_UAS_ (15530:12287) that participated before. On the other hand, the NDVI-based model shows better FVC inversion results during the booting period (R^2^ = 0.686; RMSE = 7.6%) than during the jointing period (R^2^ = 0.498; RMSE = 8.0%). This may be mainly due to the generally low FVC during the jointing period, as well as the existence of abundant mixed pixels in the UAS images with a spatial resolution of 5 cm, while the FVC during the booting period is relatively higher, and pure pixels are more dominant in the wheat field. As we all know, pixel purity is an important factor affecting the accuracy of binary models (Gao et al., 2020). This phenomenon is not only commonly observed in the estimation of winter wheat FVC but also in the estimation of FVC in soybeans (De la Casa et al., 2018), shrubs (Liu et al., 2019), and broad-leaved forests (Wang et al., 2020b). Therefore, we suggest that when using the NDVI-based model to estimate FVC, the issue of the number of mixed pixels should be carefully considered.

### 3.3 Analysis of results of different scale conversion strategies

Compared to only upscaling UAS data, the Strategy of Upscaling-Downscaling not only increases the spatial resolution of FVC from satellite pixel level to sub-pixel level but also significantly improves the prediction accuracy, especially in the wheat field boundary area. The RMSE of the fitting between the predicted results and the FVC_real_ decreased by 28.2% during the jointing period and 16.1% during the booting period. When the strategy of this method was applied to other FVC estimation models, it was found that the prediction accuracy of all models during the booting period of winter wheat increased by 14.1% to 16.5% (except for the LSTM model).

**Figure 10:**
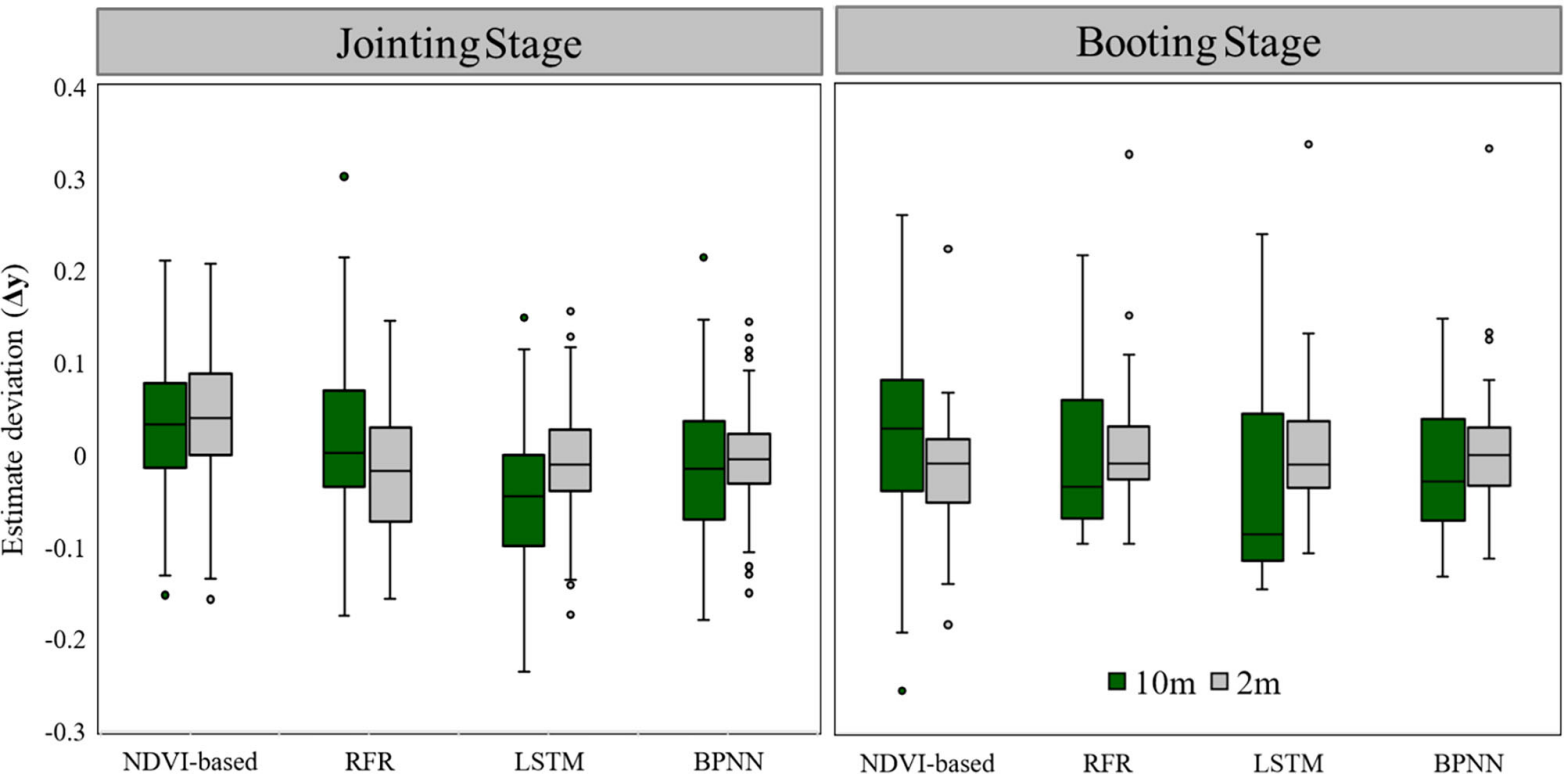
Box plots of the difference between the predicted results of different models and the reference value. (Among them, $\Delta y=FVC_{satellite}-FVC_{real}$ ).

Deep learning models can accurately approximate the complex nonlinear relationships between environmental parameters (Zhang et al., 2022), but improving their accuracy often requires a large amount of labeled training data. The amount of labeled data extracted from the 10m scale in UAS (1073 samples) is not enough to meet this requirement (**Table 4**, **Figure 10**). For the different performances of the NDVI-based model in the jointing and booting stages, the main reason is the theoretical basis of its algorithm, Beer-Lambert law and linear spectral mixture analysis, which are used to estimate the green vegetation cover or fraction cover of photosynthetic vegetation, based on the assumption that pixels are composed of only two elements: green vegetation and soil (Gao et al., 2020). However, NDVI is often influenced by soil background effects (Tang et al., 2020). Therefore, when winter wheat is in the jointing stage and FVC is around 0.50, the soil background and winter wheat are approximately evenly distributed in the study area, and changes in scale and labeled data volume will not significantly affect the estimation accuracy of the NDVI-based model. But when winter wheat is in the booting stage and FVC is around 0.85, most of the vegetation covers the ground, and using NDVI inappropriately to establish a model to estimate FVC results in a large estimation error (**Table 4**) and an overall overestimation of the estimation results (**Figure 10**), which is consistent with the previous results of using NDVI to estimate FVC (Liu et al., 2019; Tang et al., 2020). Therefore, we recommend that when ground survey data or synchronized UAS data is lacking, the NDVI-based model is still a good choice for vegetation with FVC around 0.50

**Table 4:** RMSE for fitting the predictions of different models to reference values.

Growth periods	Scale	NDVI-based	RFR	LSTM	BPNN
Jointing Stage	2m	0.080	0.079	0.065	**0.056**
	10m	0.082	0.083	0.089	**0.078**
Booting Stage	2m	**0.076**	0.073	0.074	**0.068**
	10m	**0.091**	0.085	0.129	**0.081**

The black font indicates the methods with larger FVC improvement after using the upscaling-downscaling strategy.

The traditional upscaling strategy involves establishing auxiliary models, including statistical models (De la Casa et al., 2018; Riihimäki et al., 2019; Wang et al., 2020b)and machine learning models [such as GBDT (Wang et al., 2019), RF (Zhu et al., 2019)], to obtain high-precision FVC through classification or regression of UAS data or high spatial resolution satellite data, which is then used as labeled data for FVC estimation of coarser spatial resolution data. The green box in **Figure 10** represents this traditional approach, which involves upscaling high spatial resolution data to obtain 10m FVC_satellite_; whereas our down sampling strategy in this paper involves down sampling lower spatial resolution data to obtain sub-pixel 2m FVC_satellite_. We believe that the significant improvement in accuracy resulting from the Strategy of Upscaling-Downscaling shown in **Table 4** and the gray box in **Figure 10** is primarily due to the coupling of the extensive and accurate spectral information in UAS data and the powerful learning ability of the ML model. Alvarez-Vanhard (Alvarez-Vanhard et al., 2021) categorizes such a strategy as the strongest synergistic effect of ‘data fusion’, and also point out that unmanned aerial vehicles have the potential to provide greater benefits than traditional approaches, while advancements in multi-source interoperability and machine learning will also contribute to achieving stronger synergistic effects.

**Figure 11:**
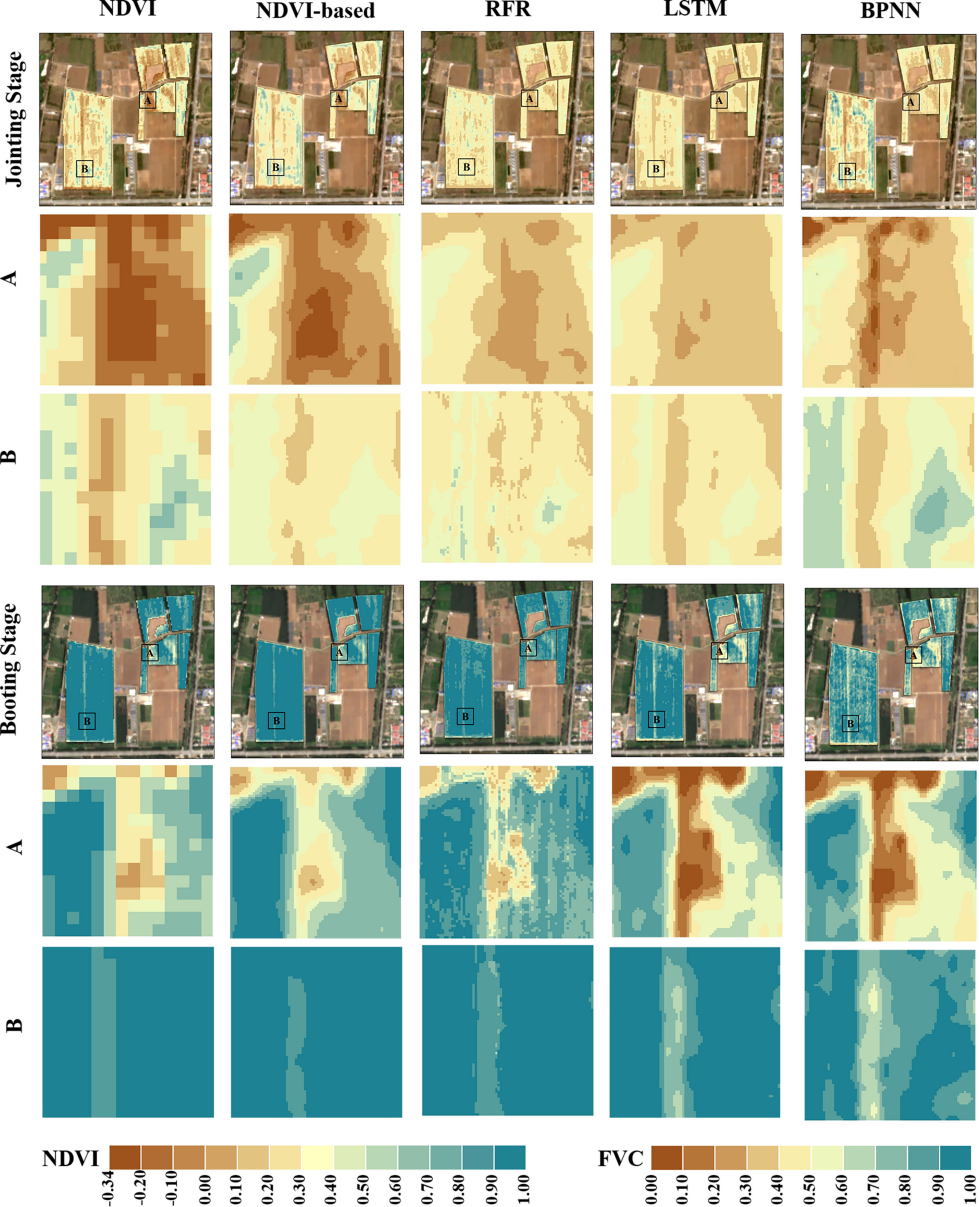
Comparison of density partition maps of FVC_satellite_ and NDVI derived from different models. The first column is a map of NDVI derived from Sentinel-2 L2A data (Spatial resolution of 10m), and the others are maps of FVC_satellite_ density splits estimated by different models (Spatial resolution of 2m); The bottom panel is an RGB plot corresponding to the growth period (Sentinel-2 L2A data).

**Figure 11** shows the estimated FVC of the four models after using the proposed strategy for two growing periods compared to the NDVI maps. The pathways and surrounding wheat fields are represented by the two black rectangular patches A and B respectively. As can be observed, spatial ambiguity is produced when the NDVI-based FVC mapping is resampled from 10m to 2m pixel size. At the Jointing Stage, some wheat fields along the field path had higher NDVI values that were more vulnerable to non-vegetation spectra and tended to be underestimated. In contrast, when the winter wheat cover level is generally high at the Booting Stage, the NDVI values of the pixels at the field path locations are more likely to be affected by the vegetation spectra, and the FVC at the wheat field boundary locations tends to be overestimated.

**Figure 12:**
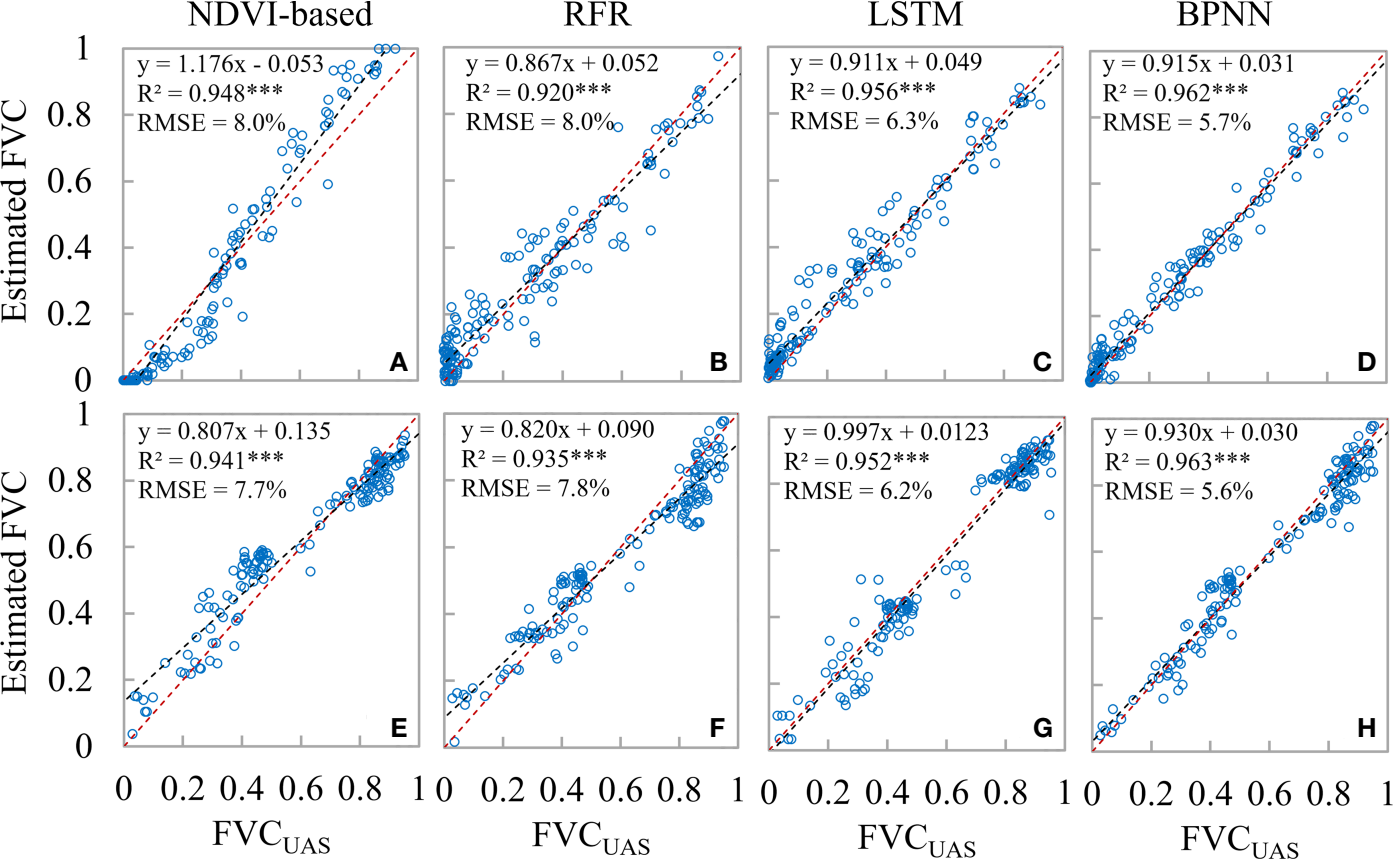
Scatterplots of estimated FVC for field boundary locations and intermediate locations under different strategies; **(A–D)** are field boundary locations; **(E–H)** are intermediate field locations.

The FVC scatter plots for the wheat field boundary and intermediate sample locations obtained using various techniques are displayed in **Figure 12**. The estimation accuracy was evaluated by randomly choosing 150 FVC_UAS_ points from the middle and border locations of the wheat field (the boundary location is defined as the area within a 5 m radius of the boundary line). The majority of the FVC-less than-0.2 sites in the NDVI-based model were situated below the 1:1 line, indicating that the model’s estimation resulted in an underestimating. At various FVC levels, the machine learning system consistently estimates the value without appreciable overestimation or underestimation. The RMSE-UAS findings based on NDVI, RFR, LSTM, and BPNN are 7.8%, 7.8%, 6.2%, and 5.6%, respectively, as shown in **Figure 6**. The RMSE in the center of each model is rather near to its respective RMSE-UAS, as seen in **Figure 12**. As a result, we think that the NDVI-based model’s higher RMSE at the boundary location (8.0%) pulls up the RMSE at the middle position (7.7%), resulting in an RMSE with an overall estimation accuracy of 7.8%. It indicates that the BPNN model can greatly increase the precision of wheat field boundary estimates. Implementing the Upscaling-Downscaling technique provides critical support for precise FVC estimates at the boundary regions of wheat fields using the BPNN model owing to the support of UAS imagery that offers Wheat coverage information with higher spatial detail. In wheat fields with high geographical diversity, such as family farms, river areas, urban or rural peripheries, etc., this is essential for accurately measuring vegetation coverage.

## 4 Conclusions

Fractional vegetation cover (FVC) is a critical trait that characterizes the growth status of crops and is of great interest in crop breeding and precision management. Here, we demonstrated a novel methodology of integrating UAS and satellite imagery for accurate estimation of winter wheat FVC.

(1) A random forest regression model with the support of limited ground reference data can obtain a high-precision FVC_UAS_ (RMSE = 0.054). By substituting UAS for field measurements, more abundant remote sensing information was incorporated into the FVC_satellite_ estimation. Moreover, with the powerful learning ability of the BPNN model, more accurate FVC estimation over larger areas can be achieved. Validation with an independent dataset showed that the RMSE of the BPNN model (0.059) was 11.9% to 25.3% lower than that of other commonly used FVC estimation models. In addition, the stable estimation can be achieved with only 20,000 label data for the BPNN model.

(2) Compared to the strategy of only upscaling UAS data, the strategy of both upscaling and downscaling UAS and satellite data simultaneously not only obtained sub-pixel FVC but also improved accuracy, especially on the border of the wheat field. Four commonly used FVC estimation models estimated winter wheat FVC during Jointing and Booting stages separately using the Strategy of Upscaling-Downscaling, achieving a maximum reduction of 28.2% and 16.5% in RMSE, while the traditional NDVI-based model was even able to achieve the most significant reduction in RMSE during the booting stage when FVC is higher. More importantly, the Strategy of Upscaling-Downscaling provided sub-pixel FVC, which enriched the spatial heterogeneity information.

The results demonstrate that the integration of UAS and satellite imagery using machine learning algorithms can achieve an accurate estimation of winter wheat FVC. Therefore, we recommend collecting ‘Ground-UAS-Satellite’ data synchronously during fieldwork and crop parameter inversion. Furthermore, the proposed method framework can be applied to estimate other surface parameters and synergistically utilize multiple sources of data.

## Data availability statement

The raw data supporting the conclusions of this article will be made available by the authors, without undue reservation.

## Author contributions

The experiment was mainly conceived and designed by BZ. SY, RY, CL, JH, ShaL, EC, and ZL performed the experiments. BZ, SY, ShaL, and DP analyzed the data. The algorithm development was mainly accomplished by BZ, SY, and ShaL. SY wrote the manuscript and BZ made very significant revisions. ShaL and DP also read and improved the final manuscript. All authors contributed to the article and approved the submitted version. 
